# HIV Drug Resistance Mutations (DRMs) Detected by Deep Sequencing in Virologic Failure Subjects on Therapy from Hunan Province, China

**DOI:** 10.1371/journal.pone.0149215

**Published:** 2016-02-19

**Authors:** Xi Chen, Xiaobai Zou, Jianmei He, Jun Zheng, Jennifer Chiarella, Michael J. Kozal

**Affiliations:** 1 Hunan Provincial Center for Disease Control and Prevention, Changsha, China; 2 Yale School of Medicine, New Haven, United States of America; Institut Pasteur of Shanghai, CHINA

## Abstract

**Objective:**

Determine HIV drug resistance mutations (DRMs) prevalence at low and high levels in ART-experienced patients experiencing virologic failure (VF).

**Methods:**

29 subjects from 18 counties in Hunan Province that experienced VF were evaluated for the prevalence of DRMs (Stanford DRMs with an algorithm value ≥15, include low-, intermediate and high-level resistance) by both Sanger sequencing (SS) and deep sequencing (DS) to 1% frequency levels.

**Results:**

DS was performed on samples from 29 ART-experienced subjects; the median viral load 4.95×10^4^ c/ml; 82.76% subtype CRF01_AE. 58 DRMs were detected by DS. 18 DRMs were detected by SS. Of the 58 mutations detected by DS, 40 were at levels <20% frequency (26 NNRTI, 12 NRTI and 2 PI) and the majority of these 95.00% (38/40) were not detected by standard genotyping. Of these 40 low-level DRMs, 16 (40%) were detected at frequency levels of 1–4% and 24 (60%) at levels of 5–19%. SS detected 15 of 17 (88.24%) DRMs at levels ≥ 20% that were detected by DS. The only variable associated with the detection of DRMs by DS was ART adherence (missed doses in the prior 7 days); all patients that reported missing a dose in the last 7 days had DRMs detected by DS.

**Conclusions:**

DS of VF samples from treatment experienced subjects infected with primarily AE subtype frequently identified Stanford HIVdb NRTI and NNRTI resistance mutations with an algorithm value 15. Low frequency level resistant variants detected by DS were frequently missed by standard genotyping in VF specimens from antiretroviral-experienced subjects.

## Introduction

At the end of 2013, Hunan province which is located in south central China had a population of 68 million. Since the first case of HIV was diagnosed in Hunan in 1992, 19,661 HIV /AIDS cases have been reported through 2013 with 5,871 AIDS related deaths. The primary HIV transmission modes in this region are injection drug use (IDU) and sexual contact. By the end of 2013, 9,594 HIV positive patients were under care and receiving free Antiretroviral Therapy (ART) which is supported by the government in Hunan Province, China. Initial choices for first line antiretroviral therapy consist of triple therapy selected from Stavudine (d4T), Lamivudine (3TC), Zidovudine (AZT), Tenofovir (TDF), Efavirenz (EFV) and Nevirapine (NVP), EFV, AZT, d4T, 3TC and NVP are generically produced in China. Lopinavir/ritonavir (LPV/r) a second-line drug has not yet been widely used in Hunan Province. Epidemiological surveys have revealed that China is currently one of the countries in which a wide range of HIV-1 subtypes and CRFs are cocirculating [1]. A recent Hunan Province molecular epidemiology survey (2009–2013) revealed that 4 HIV-1 subtypes, CRF_01AE, CRF07_BC, B and C are circulating in Hunan Province with CRF_01AE being the dominant subtype (more than 80%) [2–6].

The sensitive and accurate detection of drug resistance mutations (DRMs) is very important to the proper diagnosis and treatment of HIV-infected persons. Infection with DRMs can lead to ART failure and is associated with increased mortality [7 –10]. Standard HIV genotyping using Sanger sequencing (SS) methods may not detect all resistant viral variants. The SS methods in clinically approved HIV genotypic resistance assays typically can detect dominant viral variants (resistant variants level ≥esi frequency) but will miss mostly low-level variants with mutations (<20%) [7–11]. Recent studies have demonstrated that some low level DRMs (> 1%) can become dominant variants rapidly under drug selected pressure and lead to virologic failure (VF) [10, 12–16]. However, these low level DRMs can be detected by more sensitive technologies like deep sequencing (DS) [17–24]. In this study, we define ‘‘low-level” DRMs as mutations detected at <20% frequency levels of the viral quasi-species by DS. There is little data on the prevalence of low level HIV variants with drug resistance mutations in non B/C HIV subtypes and very few studies investigating low-level drug-resistant variants in subjects experiencing virologic failure on first line ART in China.

The objective of this study was to determine the prevalence of Stanford HIVdb and DRMs at both low and high levels in ART-experienced VF subjects by DS and by standard HIV genotyping.

## Materials and Methods

### Ethics Statement

The research protocol, approved by the relevant institutional review boards or independent ethics committees was conducted in accordance with standards for the protection of patient safety and welfare and in compliance with Good Clinical Practices and the principles of the Declaration of Helsinki and its amendments. The study was conducted at two clinical sites, in Hengyang City and Changsha City Hunan Province, after it was approved by the local Ethics committee: Hunan Provincial Center for Disease Control and Prevention Human Investigative Committees/IRBs. At each site, clients receive comprehensive evaluation and ART when indicated. According to “National Free AIDS ARV guideline” (China, 2012), all participants who receive treatment from AIDS ARV that support by Chinese government, have to perform viral load testing and genotypic testing, which will be used for this study. Verbal informed consent will be given by the doctors at the two clinical sites who are responsible for the AIDS patients, and their names will be kept on a list with the doctors’ signature. The study and the verbal consent procedure were approved by a single IRB, and its name is “the Hunan Provincial Center for Disease Control and Prevention Human Investigative Committee”.

### Patients

The Chinese national free ARV treatment policy guidelines recommends that patients on ART should have an HIV viral load test at least once a year [25]. If the viral load is >1000 copies/ml a HIV drug resistance genotypic test (Sanger Sequencing based) should be performed. A total of 29 subjects with AIDS from 18 counties in Hunan Province were enrolled in the study. Primary inclusion criteria: 1) HIV-infected; 2) on ART more than 12 months; 3) a minimum of 18 years of age; 4) a plasma HIV RNA ≥10,000 copies/mL with any CD4+ lymphocyte count.

### RNA extraction, amplification and Sanger sequencing

RNA was isolated from 140 ul of plasma using the QIAmp Viral RNA mini kit (Qiagen, Germany), in accordance with the manufacturer’s protocol, and eluted to a 60 uL suspension. Approximately 1.4×10^3^ bp pol fragments (HXB2 positions 2028–3462) were amplified by nested reverse transcriptase polymerase chain reaction (nested RT-PCR) using RT-PCR kits (Takara, Dalian, China) and Taq PCR Mastermix (Tiangen, Beijing, China), the *pol* region including the entire protease gene and partial polymerase gene for genotyping and DRM analysis.

The results from sequencing were aligned and assembled manually into contiguous sequences using Contig Express Project, a component of the Vector NTI Suite 6 software. To determine drug-resistant mutations and amino acid polymorphisms, each sequence was analysis in the Stanford HIV Drug Resistance Database (http://hivdb.stanford.edu).

### Deep sequencing (454) analysis

The viral RNA was isolated from 140 ul of plasma using the QIAmp Viral RNA mini kit (Qiagen, Germany) with 2 ug of the included polyA-RNA carrier, and were sent to Application support center (Roche Applied Science, Asia Pacific) for DS. The laboratory was given viral load information but was blinded to the standard sequencing results and clinical data. There were four primers sets that were used to amplify the HIV POL region, include protease (PR) and reverse transcriptase (RT) genes. The fusion primers containing the Roche 454 amplicon adaptor sequences, multiplex identifier (MID) tags on both forward and reverse primers. Following PCR amplification, all PCR products were purified using an AMPure XP (Agencourt) bead clean-up for twice, then quantitated using the Quant-iT PicoGreen dsDNA Assay Kit (Invitrogen Carlsbad, CA, USA), according to the Protocol for Amplicon Sequencing of HIV RT and PR(454 Life Sciences, Roche)[26]. Then all amplicons were pooled together at equimolar ratios, for the emulsion PCR which was performed according to GS Junior emPCR amplification method manual (454-Roche, USA). After the enrichment of the emulsion PCR products, the picotiter plate (PTP) was prepared and 5.00×00^5^ enriched beads were deposited into each well of PTP and pyrosequenced from both ends (forward and reverse) by GS Junior System (454 Life Sciences, Roche, Branford, CT, US).

### Drug Resistance Mutation Analysis

The sequencing results were analyzed using the GS Amplicon Variant Analyzer (AVA) software (Roche). The subtype and DRMs was performed by AVA. Ultra deep sequencing was performed as described in previous studies and the level of DS variant detection has been previously reported [27–31]. For the purpose of discussion in this paper, we reported a variant detection limit of >1% as resistant variants at this level have been shown to be clinically relevant [10, 32–34]. In addition, the level of >1% was chosen as the 454 platform used in this study has demonstrated high intralaboratory and interlaboratory consistency in the detection of mutants present at a frequency between 1 and 10% in a large multicentre evaluations [35–37]

The analysis for DRMs in this study was performed using Stanford HIV Drug-Resistance Database and all mutations with a resistance score of e 15 for any drug were considered (http://hivdb.stanford.edu) [38]. For the purpose of discussion in this paper, low-level DRMs mutations were defined as mutations detected at <20% level of the viral quasispecies, whereas high-level DRMs were those detected at ≥20%, and the statistical analysis was performed using SPSS computer software (SPSS, Version 13.0, SPSS, Inc., Chicago, IL). Findings with ***P*** < 0.05 were considered statistically significant.

## Result

All 29 subjects had experienced virologic failure on ART with a median HIV viral load of VF of 4.95×10^4^ c/ml (range: 1.41×10^4^−8.66×10^5^ copies/ml). The median CD4 cell count at VF was 178 cells/mm^3^ (range 1 to 579 cells/mm^3^); 82.76% (24/29) of subjects were infected with HIV subtype CRF01_AE, 10.34% (3/29) B and 6.90% (2/29) were subtype C. Among these 29 subjects, 22 (75.86%) were male and 7 (24.14%) were female. HIV transmission risk factors reported: 20 subjects (68.97%) via heterosexual sex, 8 (27.59%) IDU, and 1 (3.45%) MSM. All were adults with a mean age of 41.42 years of age (range: 24 to 75).

Of the 29 subjects, 44.83% (13/29) had been treated in 2012, 24.14% (7/29) in 2011 and 31.03 (9/29) were started on ART before 2010; 44.83% (13/29) subjects were on the first line regimen NVP+3TC+AZT, 41.38% (12/29) on EFV+3TC+AZT, with 2 subjects on 3TC+AZT+LVP/r, 1 on EFV+3TC+TDF and 1 received NVP+3TC+TDF.

All 29 subjects had HIV genotyping performed by SS and DS. Of the 29 subjects, 37.93% (11/29) did not have any DRMs (algorithm value ≥15) identified by SS or DS. Of these 11 subjects, 6 were receiving NVP+3TC+AZT, 4 on EFV+3TC+AZT and 1 on 3TC+AZT+LPV/r. Eighteen of 29 (62.07%) subjects had viral variants with a DRM to any PI, N(t)RTI and/or NNRTI drug (algorithm value ≥15) at levels >1% by DS and/or SS. Nine subjects (31.03%) had DRMs detected by both SS and DS, and 9 (31.03%) had DRMs detected only by DS.

A total of 58 DRMs were identified in the 18 subjects by either of the two methods; 62.07% (36/58) were NNRTI mutations: K103N(8), V108I(5), G190A/E(5), Y188C/H/L(4), V106A/M(2), Y181C/V(3), F227L(2), E138G/Q(2), P225H(2), K101E(2), A98G(1); 34.48%(20/58) were NRTI: M184V(5), T215F(3), M41L(2), K65R/N(2), D67N(2), L74V(2), V75M(2), K70R(1), K219(1); and only 3.4% (2/58) were PI mutations: LV32I and M46L (Table 1). Only 18 DRMs were detected by SS. 13 (68.42%, 13/19) NNRTI mutations: K103N(6), Y181C/V(2), G190A(2), Y188H(1), K101E(1), T215F(1); 5 NRTI mutations: M184V(3), K65R(1), D67N(1); and two PI mutation were not detected by SS. Of the DRMs detected by SS, Seventeen (94.4%) of the 18 DRMs detected by SS were also detected by DS. Fifty seven of the 58 DRMs were detected by DS. One DRM detected by SS was not detected by DS, mutation levels ranged from 16.2~100.0% (Table 1). Low-level DRMs (<20%) were detected in all 18 subjects by DS and were unrecognized in 16 subjects by SS (Table 2).

**Table 1:** HIV drug resistance mutations detected by standard Sanger sequencing (SS) and by Deep sequencing (DS).

NO.	VL(COPIES/ML)	TREATMENT REGIMEN	SUBTYPE	PROTEASE (% ABUNDANCE BY DS) MUTATIONS		REVERSE TRANSCRIPTASE (% ABUNDANCE BY DS)	
				by DS only	by both SS and DS	by SS only	by DS only
1	26356	NVP+TDF+3TC	AE		K103N (16.17%)		L74V (1.44%), V108I(15.16%), F227L(2.04)
4	33645	EFV+3TC+AZT	B				Y188C (2.45%)
9	559474	EFV+3TC+TDF	B				K103N (14.97%)
11	29299	NVP+3TC+AZT	AE		K65R (28.08%, D67N (34.53%), Y181C (98.77%), Y188H (100%)		M41L (15.99%, L74V (2.2%), T215F (12.28%), E138G(12.14%), A98G (18.52%), K103N (16.17%), V106M (15.16%), V108I (10%), G190A(50.1%), F227L (2.04%)
12	75401	EFV+3TC+AZT	AE				M184V (7.52%), T215Y (6.64%), Y188L (7.52%)
14	38072	NVP+3TC+AZT	AE		K103N (98.91%)		D67N (1.44%), V108I (1.63%), G190A (2.74%), P225H (3.92%)
15	68852	EFV+3TC+AZT	AE		M184V (26.84%), K103N (97.46%)	G190A	V75M (1.72%)
18	393742	NVP+3TC+AZT	AE		M184V (91.55%), K103N (99.22%)		K70R (15.05%), V108I (16.43%), P225H (18.21%)
19	440099	NVP+3TC+AZT	C	M46L(2.68%)			
20	43792	3TC+AZT+LPV/r	AE		K103N (88.99%)		
21	30706	EFV+3TC+AZT	AE		G190A (13.45%), K101E (99.18%)		V108I (17.16%)
22	865605	EFV+3TC+AZT	AE				G190E (5.09%)
29	59874	EFV+3TC+AZT	AE		Y181V (42.08%)		M184V (9.44%), Y188H (8.71%)
30	370747	NVP+3TC+AZT	AE		M184V (100%), T215F (87.97%), K103N (99.72%)		M41L (3.48%), V75M (29.92%), E138Q (2.36%)
31	93200	NVP+3TC+AZT	AE				Y181C (1.2%)
33	49500	EFV+3TC+AZT	B				K65N (6.01%),
34	98000	EFV+3TC+AZT	AE				V106A (2.43%)
35	92200	NVP+3TC+AZT	AE	V32I (14.02%)			K101E (1.65%), G190A (1.39%)

Note. Stanford HIVdb algorithm≥15.

**Table 2:** Prevalence of ≥1% DRMs by Stanford HDRM (algorithm value ≥15).

DRMs class	Low-level mutations(<20%)		High-level mutations(≥20%)	
	SS	DS	SS	DS
NNRTI	2	26	9	10
NRTI	0	12	6	7
PI	0	2	0	0
ANY	2	40	15	17

SS—Sanger Sequencing; DS Deep Sequencing

Of the 57 DRMs detected by DS, 17 DRMs were at levels ≥20% and 40 were low-level mutations (<20%) detected by DS: 26 were NNRTI DRMs, 12 NRTI DRMs and 2 PI DRMs; the majority of these (95.00%, 38/40) were not detected by standard SS genotyping. SS detected 15 of 17 (88.24%) DRMs at levels ≥ 20% that were detected by DS. Of the total 40 low-level DRMs, 16 (40%) were detected at levels 1–4% and 24 (60%) at levels of 5–19%. The low-level DRMs mutations were detected for all three major antiretroviral classes used in Hunan Province; 10 of 29 (34.48%) subjects harbored low-level DRMs that predicted resistance to 2 antiretroviral class, nine (31.03%) to one class and no one had three class resistance (Table 2).

Factors associated with the detection of DRMs are listed in Table 3. The only variable associated with the detection of DRMs was ART adherence—missed doses in the prior 7 days [25]^]^ all patients that reported missing a dose in the last 7 days had DRMs detected by DS (Table 4). There were 9 subjects that reported missing a dose in the last 7 days and all had DRMs detect by DS with 4 having only low-level variants. The average HIV viral load for the 9 subjects with poor adherence and DRMs was 1.78×10^5^ copies/ml versus the 20 subjects with good adherence was 112,867 copies/ml (P = 0.427). The average HIV viral load for the 11 subjects without DRMs and good adherence was 4.45×10^4^ copies/ml whereas the 18 subjects with DRMs was 1.87×10^5^ copies/ml (P<0.05).

**Table 3:** Factors associated with incidence of DRMs.

Variable	Mutation identified by DS (*n* = 18)	No mutations identified by DS (*n* = 11)	Overall(*N* = 29)	*P*
Regimen				
NVP+3TC+AZT	7(38.9%)	6(54.5%)	13	0.941
EFV+3TC+AZT	8(44.4%)	4(36.4%)	12	
LPV/r +3TC+AZT	1(5.6%)	1(9.1%)	2	
NVP+3TC+TDF	1(5.6%)	0	1	
EFV+3TC+TDF	1(5.6%)	0	1	
CD4 abs count(cells/ mm^3^)				
0≤199	9(50.0%)	6(54.5%)	15	1.00
≥200	9(50.0%)	5(45.5%)	14	
WHO Stage				
I	9(50.0%)	4(36.4%)	13	0.901
II	4(22.2%)	3(27.3%)	7	
III	2(11.1%)	2(18.2%)	4	
IV	3(16.7%)	2(18.2%)	5	
Route of transmission				
IDU	5(27.8%)	3(27.3%)	8	0.503
Heterosexual	13(72.2%)	7(63.6%)	20	
MSM	0(0.0%)	1(9.1%)	1	
Recently 7 days number of doses missed				<0.05
0	9	11	20	
≥1	9	0	9	

**Table 4:** DRMs by DS and Recent 7 days ART adherence.

No.	VL(copies/ml)	> 1% FOR STANFORD HDRM by DS(algorithm value ≥15)	Recently 7 days number of doses missed
1	26356	L74V (1.44%), K103N (16.17%), V108I (15.16%), F227L (2.04%)	4
2	47344	none	0
3	23491	none	0
4	33645	Y188C (2.45%)	0
5	35449	none	0
6	37347	none	0
8	37414	none	0
9	559474	K103N (14.97%)	2
11	29299	M41L(15.99%, K65R (28.08%, D67N(34.53%), L74V (2.2%, T215F (12.28%%), E138G (12.14%), A98G(18.52%), K103N(16.17%), V106M(15.16%), V108I(10%), Y181C(98.77%), Y188H(100%), G190A(50.1%), F227L(2.04)	4
12	75401	Y184V (7.52%), T215Y (6.64%), Y188L (7.52%)	0
14	38072	D67N(1.44%), K103N(98.91%), V108I(1.63%), G190A(2.74%), P225H(3.92%)	4
15	68852	V75M(1.72%), 184V (26.84%), K103N (97.46%)	0
18	393742	K70R (16.05%), Y184V (91.55%), K103N(99.22%), V108I(16.43%), P225H(18.21%)	4
19	440099	46L(2.68%)	0
20	43792	K103N(88.99%)	0
21	30706	G190A(13.45), K101E(99.18), V108I(17.16)	14
22	865605	G190E(5.09%)	0
24	71681	none	0
25	105749	none	0
27	33632	none	0
28	14144	none	0
29	59874	Y184V(9.44%), Y181V(42.08%), Y188H(8.71%%)	3
30	370747	M41L(3.48%), V75M(29.92%), Y184V(100%), T215F(87.97%), K103N(99.72%), E138Q(2.36%),	4
31	93200	Y181C(1.2%)	0
32	18000	none	0
33	49500	K65N(6.01%),	0
34	98000	V106A(2.43%)	0
35	92200	V32I(14.02%), K101E(1.65%), G190A(1.39%)	6
36	65000	none	0

## Discussion

The results from this study revealed that CRF01_AE is still the most common subtype in Hunan province (82.76% of patients). This finding corresponds to the data from the last Hunan Province molecular epidemiology survey (2009–2013) [2–6]. In addition to the most dominant subtype, other subtypes including B and C were found, which may indicate a more complicated and diverse trend of HIV-1 epidemiology emerging in the province. In total, ~62% of subjects experiencing VF had DRMs with a Stanford HIVdb algorithm value ≥15 detected at mutation levels >1%. Of the 58 DRMs detected, NNRTI mutations were the most common (62.07%) with 11 different codon positions represented: K103N, V108I, G190A/E, Y188C/H/L, V106A/M, Y181C/V, F227L, E138G/Q, P225H, K101E, A98G. All NNRTI mutations were found in the subjects who were failing a regimen containing nevirapine (NVP) and/or efavirenz (EFV). K103N was the most common mutation identified in this study; it is a nonpolymorphic mutation that causes high-level resistance to NVP (~50-fold reduced susceptibility) and EFV (~20-fold reduced susceptibility). Other NNRTI mutations, G190A/E, V106A/M, Y181C/V cause high level resistance to NVP and EFV. K101E and Y188C/H cause intermediate or high-level resistance to NVP and low level resistance to EFV; F227L is a nonpolymorphic mutation that usually occurs in combination with V106A and P225H is usually occurs in combination with K103N [39–44].

The major NRTI mutations identified in our study were M184V, T215F, M41L, K65R/N, D67N, L74V, V75M, K70R and K219E. M184V was detected in 5 subjects all of whom were on a treatment regimen containing lamivudine (3TC). M184V causes high-level resistance to 3TC and FTC and low-level resistance to ddI and ABC. T215F is a thymidine analog mutation (TAM) that causes intermediate/ high-level resistance to AZT and d4T and low-level resistance to TDF. T215F occurs more commonly with the Type II TAMs (D67N, K70R, and/or K219E) and in this context, it affects susceptibility to TDF, ABC, and ddI less markedly than T215Y. M41L is a TAM that usually occurs with T215Y. Together, M41L and T215Y confer high-level resistance to AZT and d4T and intermediate-level resistance to TDF. However, viruses with M41L + T215Y + M184V will exhibit intermediate-level resistance to AZT and d4T and low-level resistance to TDF [40–45]. All of the RT DRMs identified by DS except K103N had been reported in another HIV drug resistance mutation DS study in Hunan province in 2013 [6].

Protease gene DS revealed that there were no DRMs causing LPV/r drug resistance. The lack of LPV/r mutations may be due to the fact that LPV/r is a second line drug and has not yet been widely used in Hunan Province during the study period. Only 2 PI mutations were identified and both were at a low-level (<20%): V32I is a nonpolymorphic substrate-cleft mutation associated with reduced susceptibility to each PI except SQV. M46L is nonpolymorphic PI-selected mutations that reduce susceptibility to IDV, NFV, FPV, LPV and ATV when present with other mutations. M46L also reduces susceptibility to TPV. IDV, NFV, FPV, LPV and ATV were not used in Hunan province, and the presence of these variants may represent a TDR acquired in another region or country or natural polymorphisms occurring at low levels.

The only variable associated with the detection of DRMs was ART adherence—missed doses in the prior 7 days; all patients that reported missing a dose in the last 7 days had DRMs detected by DS. The lack of DRMs in patients with VF and a good adherence score requires further study.

DS allowed for the detection of greater overall drug resistance burden in subjects where 17 subjects had either DRMs detected only by DS or had additional DRMs detected by DS but not detected by SS. Our study demonstrates that low level variants harboring PI, NRTI and NNRTI DRMs are commonly unrecognized by standard SS HIV genotyping in antiretroviral-experienced subjects infected with subtype AE at time of virologic failure. DRMs at levels <20% of viral quasispecies made up 69% (40/58) of the total number of mutations detected and the vast majority (>95%) were not detected by standard SS HIV genotyping. This finding is consistent with other studies that used DS to investigated subjects infected with other subtypes experiencing VF [31, 45–46]. “It has been reported by Le and colleagues in a cohort of heavily experienced subjects failing multiple ART regimens that on average 4 additional mutations (range 1 to 10) were detected by deep sequencing compared to standard sequencing [31]. The addition of these mutations present at <20% levels occurred in 77% of subjects and conferred new resistance to at least one antiretroviral drug in 50% of subjects evaluated. Similarly, in our study, 58.6% (17/29) of patients failing ART had the mutations detected at the <20% level (Table 1). The additional mutations identified only by DS increased the resistance burden in 88% (15/17) of subjects possessing DRMs which conferred new resistance to at least one ARV.” Recent studies have demonstrated that low frequency level DRMs are clinically important, as resistant variants can grow rapidly under the selection pressure exerted by ART and lead to VF [6–13, 33] and that sensitive and accurate detection of all drug-resistant HIV strains may be very important for the proper diagnosis and treatment of HIV-infected persons.

A limitation of our study is that we had no baseline genotypic data to determine if the mutant variants pre-existed in the subjects. Further, we did not have many patients on multiple prior regimens to assess the prevalence of DRMs in subjects failing multiple regimens in China. However, the report by Le and colleagues [31] suggest that deep sequencing will identify a greater level of DRMs in heavily ART treated subjects.

In summary, our study aimed to elucidate the prevalence of Stanford HIVdb DRMs detected by DS in patients experiencing treatment failure in Hunan Province, China. ART treatment-experienced subjects from Hunan Province infected predominately with subtype AE frequently possessed low-abundance HIV variants with NRTI/NNRTI DRMs. PI mutations were rarely found and may reflect the fact that PI use is infrequent in Hunan Province. Ongoing surveillance is needed for the provinces to better understand the prevalence of resistance and how best to respond to the emergence of resistance.

## Supporting Information

S1 Table HIV drug resistance mutations detected by standard Sanger sequencing (SS) and by Deep sequencing (DS). (DOCX)

S2 Table Prevalence of ≥1% DRMs by Stanford HDRM. (DOCX)

S3 Table Factors associated with incidence of DRMs. (DOCX)

S4 Table DRMs by DS and Recent 7 days ART adherence. (DOCX)
