# Supplementary material for: HIV Drug Resistance Mutations (DRMs) Detected by Deep Sequencing in Virologic Failure Subjects on Therapy from Hunan Province, China
Source: PLoS One. 2016 Feb 19;11(2):e0149215. doi: 10.1371/journal.pone.0149215 (PMC4760947; doi:10.1371/journal.pone.0149215)
Supplement: S1 Table — (DOCX) [file pone.0149215.s001.docx]

**S1 Table. HIV drug resistance mutations detected by standard Sanger sequencing (SS) and by Deep sequencing (DS).**

| **NO.** | **VL（copies/ml）** | **Treatment regimen** | **Subtype** | **Protease (% abundance by DS) Mutations** | | **Reverse Transcriptase (% abundance by DS)** | |
| --- | --- | --- | --- | --- | --- | --- | --- |
|  |  |  |  | by DS only | by both SS and DS | by SS only | by DS only |
| 1 | 26356 | NVP+TDF+3TC | AE |  | K103N (16.17%) |  | L74V (1.44%), V108I(15.16%), F227L(2.04) |
| 4 | 33645 | EFV+3TC+AZT | B |  |  |  | Y188C (2.45%) |
| 9 | 559474 | EFV+3TC+TDF | B |  |  |  | K103N (14.97%) |
| 11 | 29299 | NVP+3TC+AZT | AE |  | K65R（28.08%, D67N (34.53%)，Y181C (98.77%), Y188H (100%) |  | M41L (15.99%, L74V (2.2%), T215F (12.28%), E138G(12.14%), A98G (18.52%), K103N (16.17%), V106M (15.16%), V108I (10%) , G190A(50.1%), F227L (2.04%) |
| 12 | 75401 | EFV+3TC+AZT | AE |  |  |  | M184V (7.52%), T215Y (6.64%), Y188L (7.52%) |
| 14 | 38072 | NVP+3TC+AZT | AE |  | K103N (98.91%) |  | D67N (1.44%), V108I (1.63%), G190A (2.74%), P225H (3.92%) |
| 15 | 68852 | EFV+3TC+AZT | AE |  | M184V (26.84%), K103N (97.46%) | G190A | V75M (1.72%) |
| 18 | 393742 | NVP+3TC+AZT | AE |  | M184V (91.55%), K103N (99.22%) |  | K70R (15.05%), V108I (16.43%), P225H (18.21%) |
| 19 | 440099 | NVP+3TC+AZT | C | M46L(2.68%) |  |  |  |
| 20 | 43792 | 3TC+AZT+LPV/r | AE |  | K103N (88.99%) |  |  |
| 21 | 30706 | EFV+3TC+AZT | AE |  | G190A (13.45%), K101E (99.18%) |  | V108I (17.16%) |
| 22 | 865605 | EFV+3TC+AZT | AE |  |  |  | G190E (5.09%) |
| 29 | 59874 | EFV+3TC+AZT | AE |  | Y181V (42.08%) |  | M184V (9.44%), Y188H (8.71%) |
| 30 | 370747 | NVP+3TC+AZT | AE |  | M184V (100%), T215F (87.97%), K103N (99.72%) |  | M41L (3.48%), V75M (29.92%), E138Q( 2.36%) |
| 31 | 93200 | NVP+3TC+AZT | AE |  |  |  | Y181C (1.2%) |
| 33 | 49500 | EFV+3TC+AZT | B |  |  |  | K65N (6.01%), |
| 34 | 98000 | EFV+3TC+AZT | AE |  |  |  | V106A (2.43%) |
| 35 | 92200 | NVP+3TC+AZT | AE | V32I (14.02%) |  |  | K101E (1.65%), G190A (1.39%) |

Note. Stanford HIVdb algorithm≥15.
