# Supplementary material for: HIV Drug Resistance Mutations (DRMs) Detected by Deep Sequencing in Virologic Failure Subjects on Therapy from Hunan Province, China
Source: PLoS One. 2016 Feb 19;11(2):e0149215. doi: 10.1371/journal.pone.0149215 (PMC4760947; doi:10.1371/journal.pone.0149215)
Supplement: S2 Table — (DOCX) [file pone.0149215.s002.docx]

**S2 Table. Prevalence of ≥1% DRMs by Stanford HDRM**

| DRMs class | Low-level mutations(<20%) | | High-level mutations(≥20%) | |
| --- | --- | --- | --- | --- |
|  | SS | DS | SS | DS |
| NNRTI | 2 | 26 | 9 | 10 |
| NRTI | 0 | 12 | 6 | 7 |
| PI | 0 | 2 | 0 | 0 |
| ANY | 2 | 40 | 15 | 17 |

*SS – Sanger Sequencing; DS Deep Sequencing
