# Supplementary material for: HIV Drug Resistance Mutations (DRMs) Detected by Deep Sequencing in Virologic Failure Subjects on Therapy from Hunan Province, China
Source: PLoS One. 2016 Feb 19;11(2):e0149215. doi: 10.1371/journal.pone.0149215 (PMC4760947; doi:10.1371/journal.pone.0149215)
Supplement: S3 Table — (DOCX) [file pone.0149215.s003.docx]

**S3 Table. Factors associated with incidence of DRMs**

| Variable | Mutation identified by DS (*n*=18) | No mutations identified by DS (*n*= 11) | Overall(*N*=29) | *P* |
| --- | --- | --- | --- | --- |
| Regimen |  |  |  |  |
| NVP+3TC+AZT | 7(38.9%) | 6(54.5%) | 13 | 0.941 |
| EFV+3TC+AZT | 8(44.4%) | 4(36.4%) | 12 |  |
| LPV/r +3TC+AZT | 1(5.6%) | 1(9.1%) | 2 |  |
| NVP+3TC+TDF | 1(5.6%) | 0 | 1 |  |
| EFV+3TC+TDF | 1(5.6%) | 0 | 1 |  |
| CD4 abs count(cells/ mm^3^) |  |  |  |  |
| 0≤199 | 9(50.0%) | 6(54.5%) | 15 | 1.00 |
| ≥200 | 9(50.0%) | 5(45.5%) | 14 |  |
| WHO Stage |  |  |  |  |
| I | 9(50.0%) | 4(36.4%) | 13 | 0.901 |
| II | 4(22.2%) | 3(27.3%) | 7 |  |
| III | 2(11.1%) | 2(18.2%) | 4 |  |
| IV | 3(16.7%) | 2(18.2%) | 5 |  |
| Route of transmission |  |  |  |  |
| IDU | 5(27.8%) | 3(27.3%) | 8 | 0.503 |
| Heterosexual | 13(72.2%) | 7(63.6%) | 20 |  |
| MSM | 0(0.0%) | 1(9.1%) | 1 |  |
| Recently 7 days number of doses missed |  |  |  | <0.05 |
| 0 | 9 | 11 | 20 |  |
| ≥1 | 9 | 0 | 9 |  |
