# Supplementary material for: HIV Drug Resistance Mutations (DRMs) Detected by Deep Sequencing in Virologic Failure Subjects on Therapy from Hunan Province, China
Source: PLoS One. 2016 Feb 19;11(2):e0149215. doi: 10.1371/journal.pone.0149215 (PMC4760947; doi:10.1371/journal.pone.0149215)
Supplement: S4 Table — (DOCX) [file pone.0149215.s004.docx]

**S4 Table. DRMs by DS and Recent 7 days ART adherence**

| No. | VL（copies/ml） | > 1% FOR STANFORD HDRM by DS(algorithm value ≥15) | Recently 7 days number of doses missed |
| --- | --- | --- | --- |
| 1 | 26356 | L74V (1.44%), K103N (16.17%), V108I (15.16%),F227L (2.04%) | 4 |
| 2 | 47344 | none | 0 |
| 3 | 23491 | none | 0 |
| 4 | 33645 | Y188C (2.45%) | 0 |
| 5 | 35449 | none | 0 |
| 6 | 37347 | none | 0 |
| 8 | 37414 | none | 0 |
| 9 | 559474 | K103N (14.97%) | 2 |
| 11 | 29299 | M41L（15.99%, K65R（28.08%, D67N(34.53%)，L74V (2.2%, T215F (12.28%%)，E138G (12.14%), A98G(18.52%), K103N(16.17%), V106M(15.16%),V108I(10%),Y181C(98.77%),Y188H(100%),G190A(50.1%),F227L(2.04) | 4 |
| 12 | 75401 | Y184V (7.52%), T215Y (6.64%)，Y188L (7.52%) | 0 |
| 14 | 38072 | D67N(1.44%),K103N(98.91%),V108I(1.63%),G190A(2.74%),P225H(3.92%) | 4 |
| 15 | 68852 | V75M(1.72%), 184V (26.84%)，K103N (97.46%) | 0 |
| 18 | 393742 | K70R (16.05%), Y184V (91.55%)，K103N(99.22%),V108I(16.43%),P225H(18.21%) | 4 |
| 19 | 440099 | 46L(2.68%) | 0 |
| 20 | 43792 | K103N(88.99%) | 0 |
| 21 | 30706 | G190A(13.45),K101E(99.18),V108I(17.16) | 14 |
| 22 | 865605 | G190E(5.09%) | 0 |
| 24 | 71681 | none | 0 |
| 25 | 105749 | none | 0 |
| 27 | 33632 | none | 0 |
| 28 | 14144 | none | 0 |
| 29 | 59874 | Y184V(9.44%)，Y181V(42.08%),Y188H(8.71%%) | 3 |
| 30 | 370747 | M41L(3.48%),V75M(29.92%),Y184V(100%),T215F(87.97%)，K103N(99.72%),E138Q(2.36%), | 4 |
| 31 | 93200 | Y181C(1.2%) | 0 |
| 32 | 18000 | none | 0 |
| 33 | 49500 | K65N(6.01%), | 0 |
| 34 | 98000 | V106A(2.43%) | 0 |
| 35 | 92200 | V32I(14.02%)，K101E(1.65%),G190A(1.39%) | 6 |
| 36 | 65000 | none | 0 |
